# Changes in plasma PLAC-1 concentration and its expression during early-mid pregnancy in bovine placental tissues – a pilot study

**DOI:** 10.1186/s12917-024-03898-z

**Published:** 2024-02-20

**Authors:** Monika Jamioł, Magdalena Sozoniuk, Jacek Wawrzykowski, Marta Kankofer

**Affiliations:** 1 Department of Biochemistry, Faculty of Veterinary Medicine, University of Life Sciences in Lublin, Akademicka Street 12, Lublin, 20-033 Poland; 2 Institute of Plant Genetics, Breeding and Biotechnology, Faculty of Agrobioengineering, University of Life Sciences in Lublin, Akademicka Street 15, Lublin, 20-950 Poland

**Keywords:** Bovine pregnancy, Placenta-specific protein 1, PLAC1, Bovine placenta

## Abstract

**Background:**

Placenta-specific protein 1 (PLAC1) is a small secreted protein considered to be a molecule with a significant role in the development of the placenta and the establishment of the mother-foetus interface. This study aimed to confirm the presence of bovine PLAC1 and to examine its profile in the placenta and plasma in the first six months of pregnancy. The expression pattern of PLAC1 was analysed by RT-qPCR and Western Blotting. Quantitative evaluation was carried out using ELISA.

**Results:**

PLAC1 concentrations in the plasma of pregnant cows were significantly higher (*p* < 0.05) than those obtained from non-pregnant animals. PLAC1 protein concentrations in the placental tissues of the foetal part were significantly (*p* < 0.05) higher than in the tissues of the maternal part of the placenta. *PLAC1* transcripts were detected in both placental tissue samples and epithelial cell cultures.

**Conclusions:**

In conclusion, the results of the present preliminary study suggest that PLAC1 is involved in the development of bovine placenta. The presence of this protein in the plasma of pregnant animals as early as the first month may make it a potential candidate as a pregnancy marker in cows. Further studies on exact mechanisms of action of PLAC1 in bovine placenta are necessary.

**Supplementary Information:**

The online version contains supplementary material available at 10.1186/s12917-024-03898-z.

## Background

A very important part of the control of bovine reproduction is the early diagnosis of pregnancy, which is now possible through several techniques, both direct and indirect. Per-rectal palpation, the oldest method, is accurate only from the 2nd month of pregnancy, therefore, ultrasound is now the more frequently used direct method in dairy cattle. Indeed, transrectal ultrasonography is a precise tool for pregnancy diagnosis that can detect pregnancy as early as 3–4 weeks after insemination, however, due to the high cost of modern equipment, its routine use is limited [1]. Also, the alternative indirect methods used to diagnose early pregnancy in cattle include the ELISA and RIA determinations of progesterone levels in serum and milk [2, 3], but these tests are subject to a certain margin of error due to dysfunction and disease within the reproductive tract [4]. The use of oestrone sulphate concentration assessment in body fluids was also considered, although this parameter can only be reliably detected at late pregnancy, after day 100 [5, 6]. Unfortunately, the ideal method for diagnosing pregnancy in cows has not yet been identified, primarily due to the limitations of sensitivity, reliability, specificity, time, ease of performing the test, and as well as cost [1]. To ensure optimal pregnancy identification, effective diagnostic methods are needed that detect pregnancy as soon as possible after conception and are cost-effective [7]. Additionally, it would be beneficial for breeders to be able to perform such tests themselves.

It is known that each stage of pregnancy is characterised by changes in protein profile and based on this specific pattern it is possible to study the mechanisms of control and regulation of the entire period of pregnancy. Current development in molecular biology and proteomics in laboratory diagnostics are enabling more profound insights into specific biomarker molecules of pregnancy that would be the earliest indicators of bovine pregnancy. Recently promising molecules include proteins, like early conception factor (ECF) [8], pregnancy–associated glycoproteins (PAGs) [9–11], interferon-tau (IFN-τ) [12, 13], and also miRNAs [14]. PAGs assay are considered the most accurate method of detecting pregnancy in the first month of pregnancy in cattle, but due to persistent serum glycoprotein levels after parturition or abortion, they may produce false positive results [10].

Placenta-specific protein 1 (PLAC1) is a protein synthesised by the trophoblastic cells, released into the extracellular matrix and secreted into the maternal bloodstream during pregnancy [15]. Studies in human and mouse models indicate that this protein plays a key role in the placentation process and one of its main tasks is to stimulate the proliferative activity of the trophoblast, which is essential for the normal growth and development of the placenta, as well as the establishment of the mother–foetus interface [16]. What is more, human studies show that circulating *PLAC1* mRNA levels rise normally during pregnancy, but its increased secretion may also be associated with pre-eclampsia, abnormal implantation and foetal damage [17]. PLAC1 is almost not detected in normal somatic cells in humans, but it is frequently presented in a wide variety of cancers, such as colorectal cancer, gastric cancer, liver cancer, osteosarcoma, endometrial serous adenocarcinoma and breast cancer [15, 18, 19]. Taking it all together, PLAC1 seems to be an interesting protein that is a key factor not only in placental cell proliferation but also in tumour cell proliferation, progression and invasion [20].

Currently, many researchers try to elucidate the exact mechanism of action of the PLAC1 protein [21], but the question of how it affects the differentiation of the different layers of the placenta has not been answered so far.

The current literature lacks a sufficient description of the PLAC1 profile and its relevance to pregnancy in cows. The aim of the study was to evaluate the PLAC1 profile in the placenta and plasma of cows and its relation to early-mid pregnancy course.

## Results

Western Blotting (WB) analyses allowed the identification of specific bands confirming PLAC1 protein expression in the examined samples. Bands of 29 ± 0.5 kDa corresponding to monomeric form of PLAC1 were detected in blood plasma and 28.1 ± 0.3 kDa in placental tissue homogenates. The original Western Blotting images including all bands, corresponding to both the mono- and possible multimeric forms of the PLAC1, are presented in Supplementary Figure S1.

**Fig. 1:**
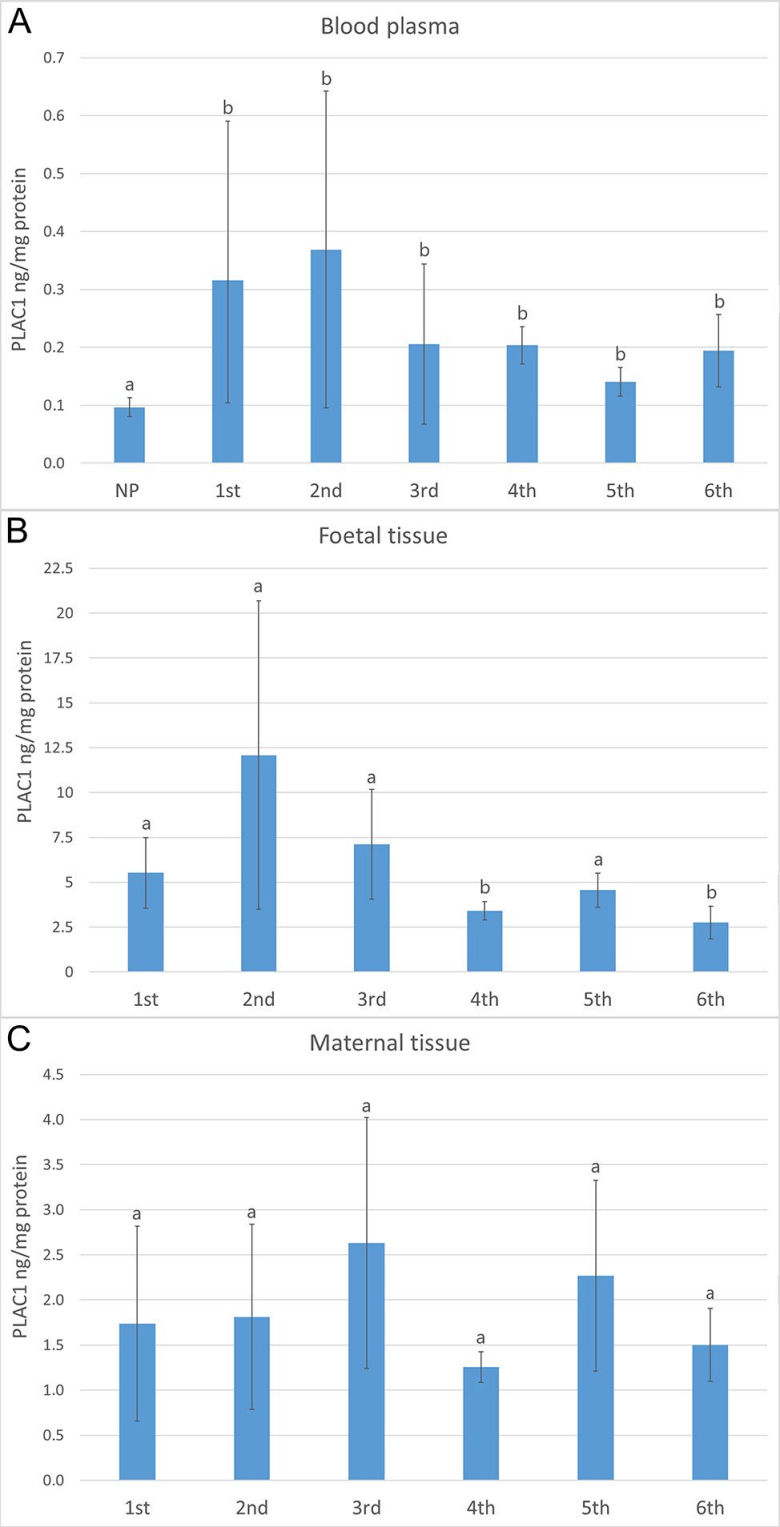
PLAC1 concentrations (ng/mg protein) in maternal blood plasma **(a)**, as well as in the bovine placental tissues – foetal **(b)** and maternal **(c)** at different gestational stages (1st – 6th month of pregnancy) determined by ELISA. NP – non-pregnant cows. Different letters represent statistical significance at *p* < 0.05

PLAC1 protein concentrations in the plasma of cows at 1st – 6th month of pregnancy (from 0.141 ± 0.025 to 0.369 ± 0.273 ng/mg protein) were significantly higher (*p* < 0.05) than the concentration of this protein in non-pregnant cows (0.097 ± 0.016 ng/mg protein). The highest PLAC1 concentrations were observed for plasma from cows in the 2nd month of pregnancy (Fig. 1a). PLAC1 protein concentrations in the placental tissues from the foetal part of the placenta (2.75 ± 0.91–12.08 ± 8.60 ng/mg protein) were significantly (*p* < 0.05) higher than those in tissues of the maternal part (1.26 ± 0.17–2.63 ± 1.39 ng/mg protein). The highest PLAC1 concentration in the foetal part of the placenta was noted in cows in the 2nd month (12.08 ± 8.60 ng/mg protein) and it was significantly higher (*p* < 0.05) compared to animals in the 4th (3.41 ± 0.51 ng/mg protein) and in the 6th (2.75 ± 0.91 ng/mg protein) months of gestation (Fig. 1b). No significant differences were observed between the examined groups of the maternal part of the placenta due to the wide spread of results within the groups from a given month of pregnancy (Fig. 1c).

Prior to *PLAC1* expression profiling, the selection of best reference genes for RT-qPCR data normalization was carried out. According to geNorm algorithm *ACTR1A* and *HDAC1* were identified as the most stable in tested material (M value = 0.4; V_2/3_ = 0.142) and subsequently used as internal controls in further analyses (Supplementary Figure S2 and S3).

*PLAC1* transcripts were detected in both placental tissue samples and cell cultures. Amplification efficiency for *PLAC1* was 107%. Regression coefficients for standard curve was 0.993. To determine the specificity of the amplification, dissociation curves were analysed. The lack of primer–dimers formation and/or unspecific product presence was confirmed by obtaining single peak on melting curves (Supplementary Figure S4).

**Fig. 2:**
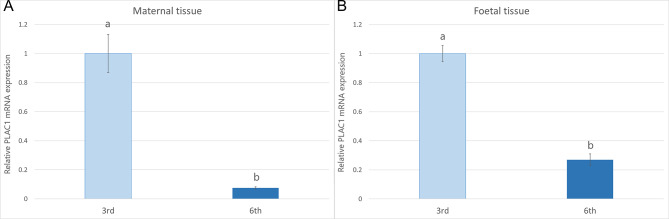
Different expression of *PLAC1* mRNA in bovine placental tissues – maternal **(a)** and foetal **(b)** in the 3rd and 6th months of pregnancy. Different letters represent statistical significance at *p* < 0.01

Relative quantification of *PLAC1* transcripts showed a significant decrease (*p* < 0.01) in its expression in both maternal and foetal parts of the placenta in the 6th month of gestation compared to the tissues from the 3rd month (Fig. 2a and b).

Furthermore, significantly higher (*p* < 0.01) *PLAC1* transcript levels were detected in the foetal part of the placenta compared to the maternal part (Supplementary Figure S5).

**Fig. 3:**
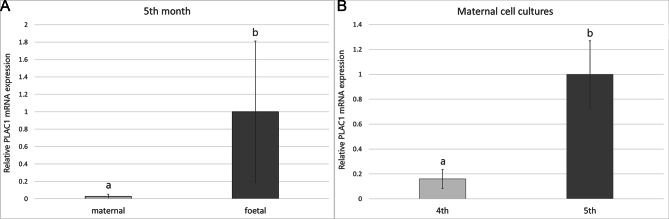
Different expression of *PLAC1* mRNA in the cell cultures – comparison of *PLAC1* mRNA expression between maternal and foetal cell cultures from the 5th month of pregnancy **(a)**, and comparison of *PLAC1* mRNA expression in the maternal cell cultures in the 4th and in the 5th month of pregnancy **(b)**. Different letters represent statistical significance at *p* < 0.01

A similar relationship was observed in the foetal cell cultures compared to the maternal epithelial cells in the 5th month of gestation, where *PLAC1* mRNA expression was higher (*p* < 0.01) in the foetal trophoblastic cells (Fig. 3a). Furthermore, upregulation of *PLAC1* transcription (*p* < 0.01) was noticed in the maternal epithelial cells in the 5th month of gestation compared to the maternal epithelial cells in the 4th month (Fig. 3b). The transcript levels in the remaining cell culture samples were not different.

## Discussion

In this study, we demonstrate for the first time the expression and concentration of PLAC1 in the placenta and plasma of healthy cows during pregnancy.

In cows, PLAC1 is a small secreted protein with a length of 179 amino acids (UniProtKB: Q32KZ6), which under physiological conditions is expressed mainly in the placenta and is substantial for proper placental development. PLAC1 protein contains two important regions - a transmembrane domain and an extracellular highly reactive ZP3 domain. ZP3 (zona pellucida 3) domain is responsible for cell adhesion and indicates the possibility of potent protein-binding interactions. This property of the ZP3 domain suggests the involvement of the PLAC1 protein in trophoblast migration and fusion with the uterine epithelium [22]. This property indicates a significant contribution of PLAC1 to the maturation of the placenta and, therefore, the normal course of pregnancy.

In the present study, the expression of PLAC1 protein and mRNA was observed both in the foetal and maternal part of the placenta. The WB results showed immune reactivity to monomeric form of PLAC1, but also to possible higher molecular weight forms corresponding to multimeric complexes. Such higher order complexes can be expected due to the tendency of the ZP3 domain in PLAC1 to form dimers, and additionally multimeric complexes of this protein do not dissociate by the sodium dodecyl sulphate used in electrophoresis [23].

PLAC1 protein concentrations in the placenta differed between the examined months of gestation. Different temporal pattern of PLAC1 may indicate specific role of the protein at particular stages of pregnancy. What is more, we found that PLAC1 protein levels were increased in the foetal placental tissues compared to the placental tissues of maternal origin, which was consistent with the RT-qPCR results. More precisely, the presented results of *PLAC1* gene expression confirm the results obtained from the quantitative analysis of PLAC1 protein – *PLAC1* expression was increased in the homogenates derived from the foetal part of the placenta. Moreover, the *PLAC1* mRNA expression was upregulated in the cell cultures originating from foetal cotyledons compared to the cells from maternal caruncles. These findings may indicate that PLAC1 plays a greater role in the foetal part of the bovine placenta. Thus, the next step should be to thoroughly investigate the specific function of this protein in the placenta of cows during pregnancy.

The exact distribution of PLAC1 protein in cows has not yet been described. The importance of this protein is mainly investigated in human and mouse models. In accordance with Fant et al. (2002), *PLAC1* mRNA expression is clearly specific for trophoblast cells during human pregnancy, without detectable expression within the stroma and maternal decidua [18]. PLAC1 protein and mRNA was also abundantly detected in the syncytiotrophoblast layer of placental villi and in smaller quantities in the trophoblast columns in early pregnancy, and also in the extravillous trophoblasts during labour [24, 25]. The abovementioned findings suggest that PLAC1 is a key molecule in the processes of human placental trophoblast syncytialization, invasion and migration. Furthermore, *PLAC1* is also plentifully expressed at the foeto–maternal interface and in different trophoblast cell subtypes in mice, and knockdown of PLAC1 negatively affects the differentiation of these cells [26].

To date, no studies confirming the importance of PLAC1 protein in cows have yet been published. However, it is worth noting that the amino acid chain length of the PLAC1 protein in cows differs from those found in humans and mice due to the shortening of the C-terminal domain [22]. Therefore, not only the properties but also a mechanism of action of this protein in cows should be particularly investigated, especially as there are differences in the placental structure between these species.

Early recognition of pregnancy is essential for both organisational and economic reasons, allowing milk production and appropriate animal nutrition to be planned and the optimum drying period to be determined [1]. A reliable diagnostic marker of pregnancy should be able to distinguish between pregnant and non-pregnant animals as early as possible.

The results obtained in the plasma of pregnant cows reflect those obtained in placental tissues from the foetal part - PLAC1 protein concentrations at 1st – 3rd months of gestation were higher than its concentrations at 4th – 6th months of gestation. This suggests that the plasma PLAC1 level profile during pregnancy is influenced by its expression in the foetal part of the placenta. What is more, in our study, significantly higher PLAC1 concentrations were detected in the plasma of pregnant cows compared to non-pregnant cows. The detected presence of PLAC1 in the plasma of non-pregnant cows may be the result of several reasons. One of the most frequently discussed causes of placental protein false positive results is an embryonic mortality occurring during the first month of pregnancy [7]. After artificial insemination in cows, embryo loss by day 42 of pregnancy ranges from 40 to 65% [27]. According to the meta-analysis on pregnancy loss incidents in dairy cattle, early embryo loss (19 to 32 days of pregnancy) affects 27% of cows [28]. Akköse (2023) reported similar blood PAGs concentrations in cows with embryonic death and healthy cattle at early pregnancy. The shifted decrease in maternal blood PAGs concentrations may be explained by the long half-life of these proteins of about 8 days [9]. Although the correct diagnosis of pregnancy may therefore be difficult, our results indicate statistically significant differences between pregnant and non-pregnant animals.

Experimental design was based on tissue availability. The relatively small sample size used in the experiments is due to the fact that it is not allowed to slaughter pregnant animals, therefore access to the material was limited. Another limitation of our study is the use of only one cell culture derived from the foetal part of the placenta. Our earlier experiments showed that while isolation and culture of maternal epithelial cells, although quite difficult, was successful in many cases [29–33], the establishment of trophoblastic cell culture proved more complex and usually failed. Among nine trophoblastic cell isolations performed, one culture was successfully maintained. This is in line with the reports of Hambruch et al. (2009) [34]. Nonetheless, inclusion of results obtained from the cell cultures has additional value due to the representation of *PLAC1* expression dedicated to specific cell types, located at the foeto-maternal interface, while homogenates show its total content without considering the distribution between placental cell layers.

## Conclusion

To conclude, we suggest considering PLAC1 as a possible marker of pregnancy in cows, but confirmation of its sensitivity and specificity requires further and more detailed studies with larger numbers of animals examining both the whole gestation period and the peri– and postnatal period. We believe that future studies should particularly focus on determining PLAC1 levels and its expression in the first month of pregnancy, as this is most relevant for the practical application. Moreover, considering the numerous reports indicating the association of high PLAC1 expression in the course of various diseases in humans, including those associated with pregnancy, a similar relationship is suspected in cows. Thus, exploring the physiological pattern of PLAC1 during pregnancy in these animals may serve in the future to investigate its profile also in the course of pregnancy-associated disorders, such as retention of foetal membranes, as their marker. Therefore, PLAC1, as an extracellular molecule, could also serve as a useful marker involved in normal placental development and foetal welfare in cows. Nevertheless, the localization of PLAC1 at the foeto–maternal interface and the exact role of PLAC1 in trophoblast differentiation in cows should be investigated.

## Materials and methods

### Plasma samples

Blood samples were collected during routine veterinary procedures in accordance with the principles of antiseptics from clinically healthy, sexually mature non-pregnant (2–8 years old; *n* = 5), and pregnant (aged 2–8 years; 1–6 month of pregnancy; *n* = 5 per each group) Polish Holstein-Fresian cows. Pregnancy age was evaluated both by the date of artificial insemination and per rectal USG. The cows were from the same farm. Efforts were made in order to obtain the most homogenous group with regard to feeding and milk production. The average milk yield was 9000 L per cow per year. All blood samples were collected on the same day in the morning. All examined animals were sampled once. Blood samples were collected from the coccygeal vein via puncture into tubes with anticoagulant and centrifuged (4 °C, 15 min, 1500 rpm). Plasma samples were collected, portioned and frozen (− 20 °C) for further analysis.

### Tissue samples

Placentas collected at a local slaughterhouse were from healthy, pregnant, Polish Holstein-Friesian cows (4–6 years old; 1–6 month of pregnancy; *n* = 4 per each group). Pregnancy age was evaluated based on the CRL of the foetus [35]. Placentomes were collected from a similar location in the uterus, manually separated into maternal and foetal parts and frozen (− 80 °C) [30, 36, 37].

#### Tissue homogenates

One-gram fragments of the tissue were homogenised (4 °C, 20 min, 6500× g) in 0.05 M phosphate buffer (pH = 7.4) containing 1% Triton X-100 and protease inhibitor cocktail (87,785, Thermo Scientific™, Warszawa, Poland) using Ultra-Turrax T 25 (Ikawerk, Janke and Kunkel Inc., Staufen, Germany). Supernatants were frozen (− 20 °C) for further analysis. Maternal and foetal parts of the placenta were analysed separately.

#### Isolation of total RNA from the tissue

Total RNA was extracted from the small piece of frozen placental tissue (0.5 × 0.5 cm) using TRIzol reagent (Invitrogen, Life Technology, Carlsbad, CA) RNA isolation protocol according to the manufacturer’s recommendations with minor modifications. Twenty µl aliquots of RNA in RNAase-free water in the presence of RiboLock RNase Inhibitor (EO0381, Thermo Fisher Scientific, Vilnius, Lithuania) were stored at − 80 °C before proceeding to further transcriptomic analyses.

### Cell cultures

Five primary epithelial cell cultures originating from bovine maternal caruncles (two from the 2nd month, two from the 4th month, and one from the 5th month of pregnancy) and one primary cell cultures originating from bovine foetal cotyledons (5th month of pregnancy) were used in this study. Cell isolation was performed according to the protocol described previously by Jamioł et al. (2021) [32]. The cells were cultured (37 °C, 5% CO_2_) in 25 cm^2^ flasks (353,108, Corning, Stryków, Poland) in a medium containing DMEM/F-12 50:50 (15-090-CVR, Corning, Stryków, Poland), 10% FBS (35-079-CV, Corning, Stryków, Poland), 100 IU/mL penicillin/100 µg/mL streptomycin (30-002-CI, Corning, Stryków, Poland) and 2 mM L-glutamine (G7513, Sigma-Aldrich, Poznań, Poland) until 80% confluence was reached. Each cell culture was prepared in 3 replicates.

#### Isolation of total RNA from the cell culture

After removing growth media, total RNA was extracted from the cell monolayer using TRIzol reagent (Invitrogen, Life Technology, Carlsbad, CA) RNA isolation protocol according to the manufacturer’s recommendations with minor modifications. Twenty µl aliquots of RNA in RNAase-free water in the presence of RiboLock RNase Inhibitor (EO0381, Thermo Fisher Scientific, Vilnius, Lithuania) were stored at − 80 °C before proceeding to further transcriptomic analyses.

### Protein concentration

The protein content of the plasma samples and homogenates was measured by the biuret method [38] using commercial assay kit (Liquick Cor - TOTAL PROTEIN 60, Cormay, Łomianki, Poland) according to the manufacturer’s instruction. The absorbance was measured at 546 nm in a DR LANGE colorimeter.

### Western blotting

Tissue homogenates and blood plasma samples derived from the 1st – 6th months of pregnancy were subjected to Western Blotting analyses. The same amounts of protein (25 µg) from plasma samples and tissue homogenates were separated in 12.5% gels by SDS-PAGE as described previously by Wawrzykowski et al. (2021) [30] with minor modifications. The separation was performed for 50 min at a constant voltage 200 V (Mini-PROTEAN® Tetra cell, Bio-Rad, Warszawa, Poland). After electrophoresis, proteins were transferred (30 min, 25 V, 1 A) to a nitrocellulose membrane using a Trans-Blot® Turbo™ Transfer System (Bio-Rad, Warszawa, Poland). The membranes were blocked with Animal-Free Blocker solution (SP-5030, Vector Laboratories, Janki, Poland) in PBST for 1 h (RT) and then incubated with 5 µl polyclonal rabbit anti-PLAC1 antibodies (6015, Pro-Sci, Flint Place Poway, CA, USA) in 7,5 ml PBST (4 °C, overnight). In the negative control, the addition of primary antibodies was omitted. After washing, the membranes were incubated with ALP-conjugated polyclonal goat anti-rabbit IgG (5 µl in 7.5 ml PBST, ab709, Abcam, Cambridge, UK) for 2 h (RT). After washing, the membranes were incubated with 1-Step™ NBT/BCIP substrate solution (34,042, Thermo Scientific™, Warszawa, Poland) until the bands were visualised. After removal of the dye, the enzyme reaction was stopped by the washing with ddH_2_O. Dried membranes were scanned with GS-710 Calibrated Imaging Densitometer (Bio-Rad, Warszawa, Poland), and analysed using Image Lab Software (Bio-Rad, Warszawa, Poland). The molecular weights of the bands were compared with the Precision Plus Protein™ Dual Color Standard (1,610,374, Bio-Rad, Warszawa, Poland) and normalised to the total protein in the same sample to correct the amount of protein loaded. Two independent experiments were performed in duplicate.

### ELISA

Tissue homogenates and blood plasma samples derived from the 1st – 6th months of pregnancy were subjected to ELISA analyses. Quantitative assessment of PLAC1 protein in undiluted plasma samples and homogenates was performed using Bovine Placenta-Specific Protein 1 (PLAC1) ELISA kits (MBS9393675, MyBioSource, San Diego, CA, USA) according to the procedure described by the manufacturer. The sensitivity of the kit was 1.0 ng/ml with the detection range of 3.12 ng/ml – 100 ng/ml. Both Intra-assay CV (%) and Inter-assay CV (%) was less than 15% (CV(%) = SD/mean × 100). Each sample was analysed in duplicate. The absorbance was measured at 450 nm in a microplate reader (RT-6900, Rayto, Shenzhen, China). The obtained results were converted into the protein content in the sample (ng PLAC1 per mg total protein).

### RT-qPCR

Placental tissues derived from the 3rd and 6th months of pregnancy and cell cultures were subjected to RT-qPCR analyses. The genomic DNA elimination and cDNA synthesis were performed using Maxima First Strand cDNA Synthesis Kit for RT-qPCR, with dsDNase (K1671, Thermo Scientific™) according to the manufacturer’s instructions. The reverse transcription was carried out on 2 µg of RNA in a final reaction volume of 20 µl. Obtained cDNA was used as a template in further transcriptomic analyses. The RT-qPCR reactions were conducted using PowerUp™ SYBR™ Green Master Mix (A25777, Applied Biosystems™). The reaction mixture of 20 µl contained 100 ng of template cDNA and 400 nM of primers (PLAC1-F: TGTACTGCCCCGCTATGTTC, PLAC1-R: TAGCATGTCTCGCCATTCCG). Primers for *PLAC1* (GenBank: BC109836.1) were designed using the Primer-BLAST tool [39]. Each reaction was carried out in three technical replicates along with a no-template control (NTC). The cycling conditions were as follows: 2 min at 50 °C, 2 min at 95 °C, 40 cycles of 15 s at 95 °C and 1 min at 60 °C. Melting curve analysis with continuous data collection from 60 to 95 °C was conducted at the end of each run in order to confirm amplification specificity and lack of primer dimer formation. The amplification efficiency of primers was determined based on the analysis of standard curve generated from serial dilution of pooled cDNA. All RT-qPCR reactions were carried out on QuantStudio™ 3 Real-Time PCR System (Applied Biosystems, USA). Obtained raw data was processed using Thermo Fisher Scientific online data analysis app (ThermoFisher Scientific, Waltham, MA, USA).

In order to ensure accurate gene expression analysis, the RT-qPCR data was normalised against *ACTR1A* and *HDAC1* reference genes. The reference genes used for data normalization were selected out of six candidates (*ACTR1A*, *CNOT11*, *HDAC1*, *RPS9*, *SUZ12*, *ZNF131*). The set of potential reference genes was chosen based on their performance in our previous study [31]. The RT-qPCR conditions for reference genes were the same as for the gene of interest, except for the amount of cDNA template used in the reactions (40 ng). Analysis of candidate genes expression stability in tested material was performed using geNorm algorithm [40].

### Statistical analysis

Differences between groups were analysed with the nonparametric Kruskal-Wallis test [41]. Pairwise comparisons were performed with the Mann-Whitney *U* tests [42]. Statistical analyses were performed with STATISTICA software (Version 13.0, StatSoft, Poland, TIBCO Software Inc., Palo Alto, CA, USA). Probability values of < 0.05 were considered statistically significant.

### Electronic supplementary material

Below is the link to the electronic supplementary material.

Supplementary Material 1: Figure S1: The original Western Blotting images confirming the presence of PLAC1 protein (a, b – blood plasma, c, d – placental tissues) at different gestational stages (1st – 6th month of pregnancy). NP – non-pregnant cows; Figure S2: Average expression stability values (M) of tested candidate reference genes according to geNorm. Genes with the lowest M-value are characterised by the most stable expression; Figure S3: Determination of the number of internal control genes required for RT-qPCR data normalization according to geNorm. The pairwise variation V_n/n+1_ < 0.15 indicates that n-number of reference genes is sufficient for obtaining reliable results and inclusion of an additional (n + 1) control gene is not required; Figure S4: Dissociation curves obtained for gene of interest (*PLAC1*) and candidate reference genes tested in this study; Figure S5: Different expression of *PLAC1* mRNA between maternal and foetal part of the placenta in the 3rd and 6th months of pregnancy in cows. Different letters represent statistical significance at *p* < 0.01.

## Data Availability

Raw data are available from the first author.

## Abbreviations

ECF: early conception factor
ECM: extracellular matrix
ELISA: enzyme-linked immunosorbent assay
IFN-τ: interferon-tau
PAGs: pregnancy–associated glycoproteins
PLAC1: Placenta-specific protein 1
RIA: radioimmunoassay
RT-qPCR: quantitative reverse transcription polymerase chain reaction
WB: Western Blotting
ZP3: zona pellucida 3
