# Supplementary material for: Changes in plasma PLAC-1 concentration and its expression during early-mid pregnancy in bovine placental tissues – a pilot study
Source: BMC Vet Res. 2024 Feb 20;20:59. doi: 10.1186/s12917-024-03898-z (PMC10877859; doi:10.1186/s12917-024-03898-z)
Supplement: Supplementary file 1 — Supplementary Material 1: Figure S1: The original Western Blotting images confirming the presence of PLAC1 protein (a, b – blood plasma, c, d – placental tissues) at different gestational stages (1st – 6th month of pregnancy). NP – non-pregnant cows; Figure S2: Average expression stability values (M) of tested candidate reference genes according to geNorm. Genes with the lowest M-value are characterised by the most stable expression; Figure S3: Determination of the number of internal control genes required for RT-qPCR data normalization according to geNorm. The pairwise variation Vn/n+1 < 0.15 indicates that n-number of reference genes is sufficient for obtaining reliable results and inclusion of an additional (n + 1) control gene is not required; Figure S4: Dissociation curves obtained for gene of interest (PLAC1) and candidate reference genes tested in this study; Figure S5: Different expression of PLAC1 mRNA between maternal and foetal part of the placenta in the 3rd and 6th months of pregnancy in cows. Different letters represent statistical significance at p < 0.01. [file 12917_2024_3898_MOESM1_ESM.pdf]

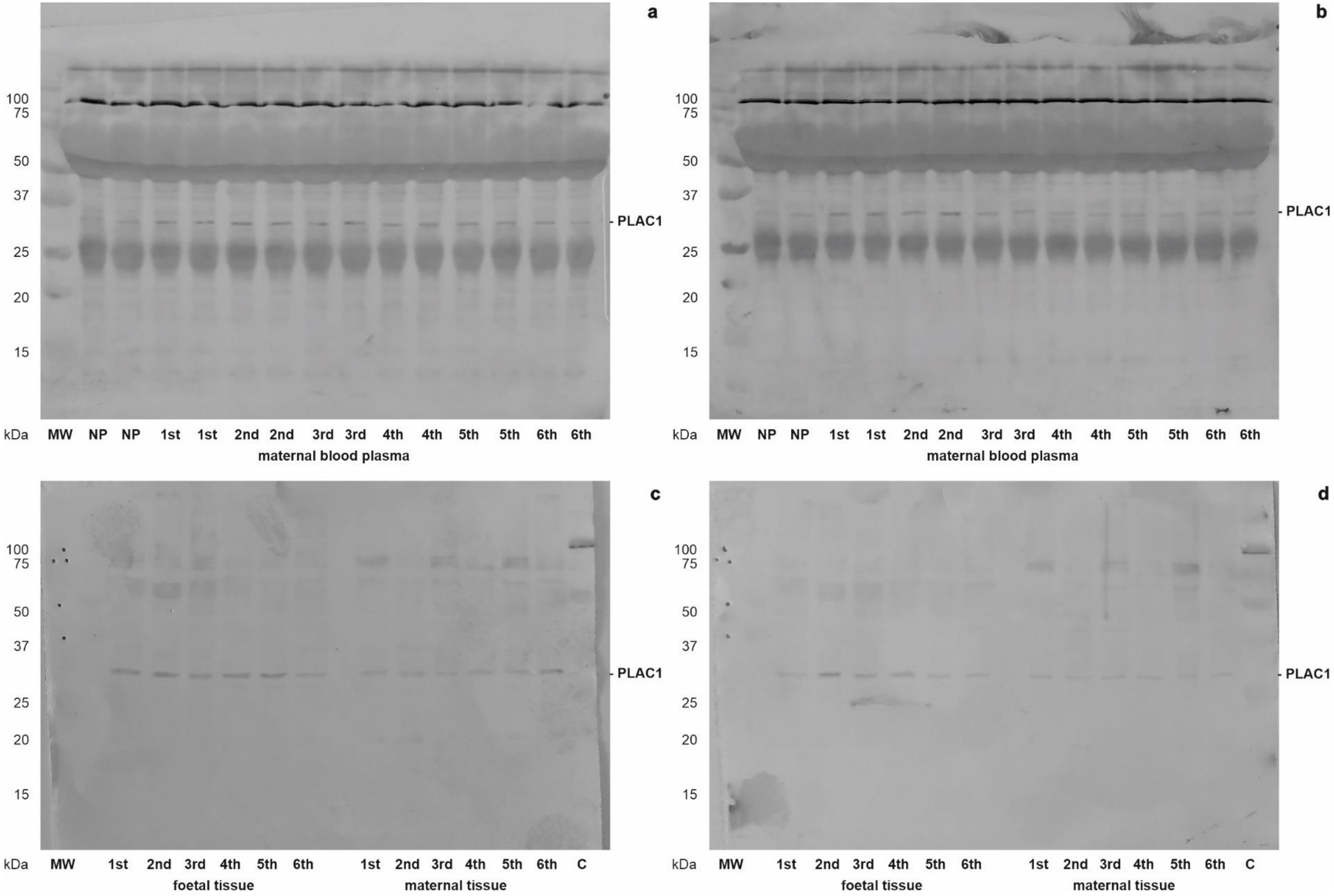

Figure S1. The original Western Blotting images (a, b – blood plasma, c, d – placental tissues).

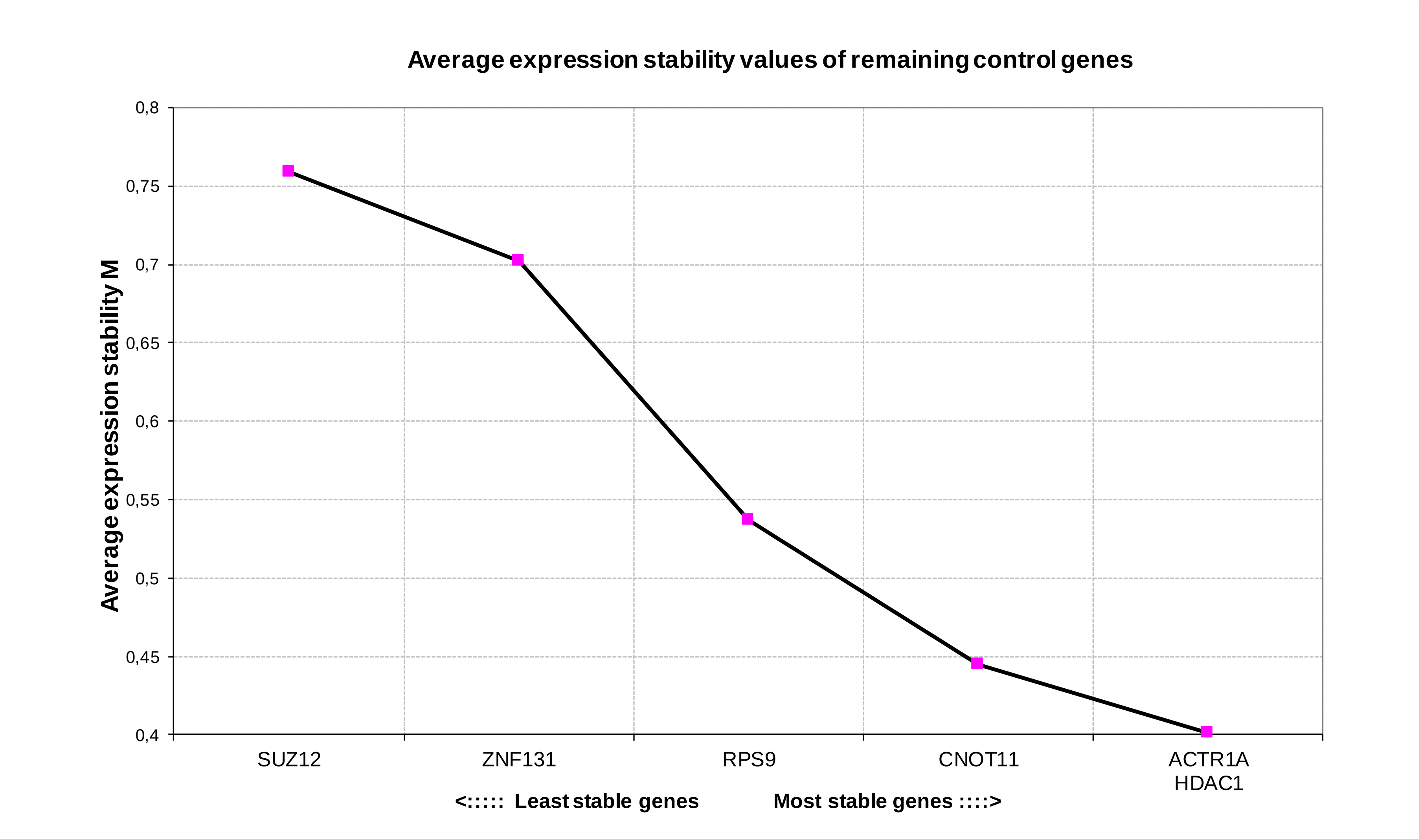

Figure S2. Average expression stability values (M) of tested candidate reference genes according to geNorm. Genes with the lowest M-value are characterized by the most stable expression.

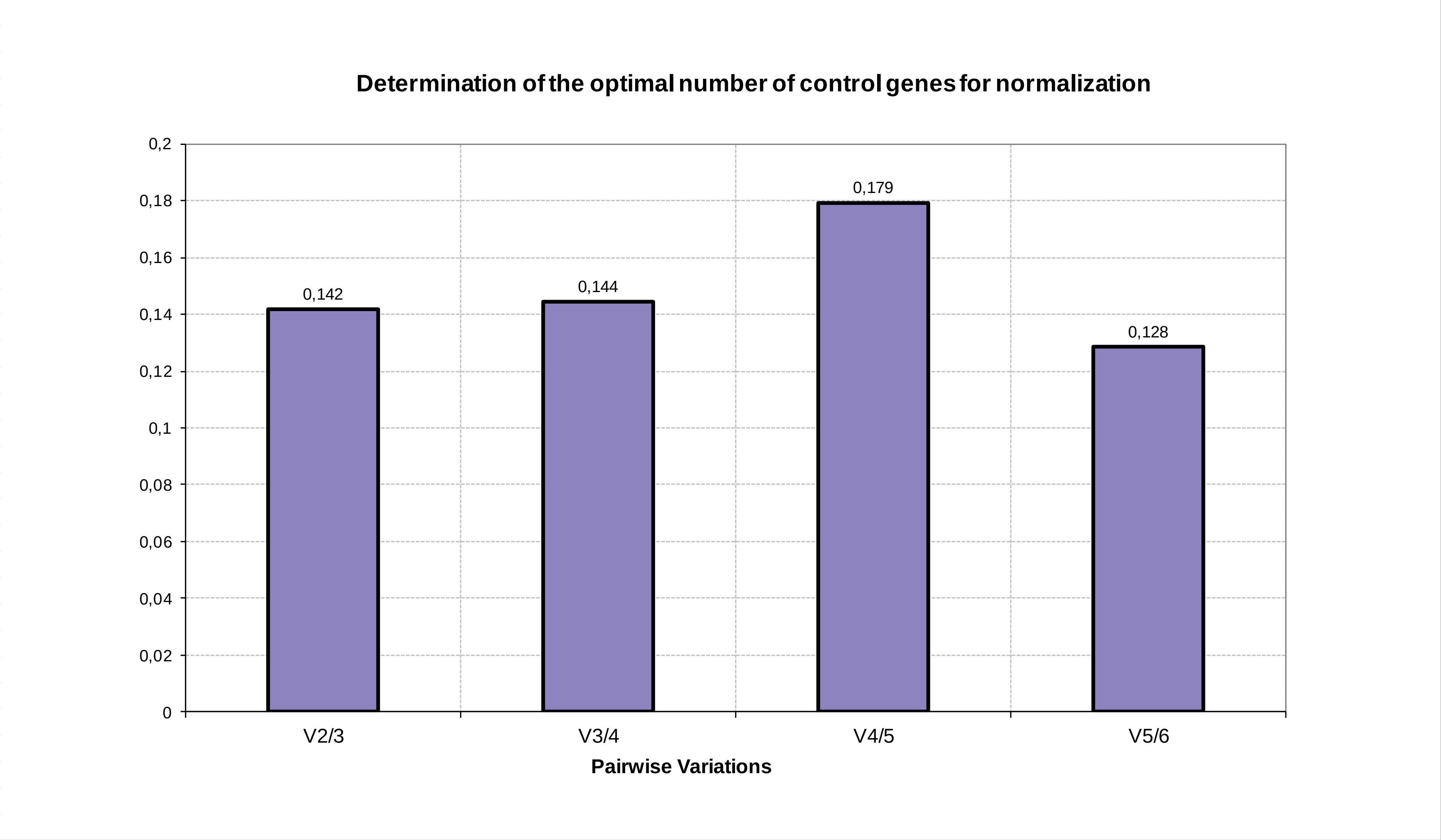

Figure S3. Determination of the number of internal control genes required for RT-qPCR data normalization according to geNorm. The pairwise variation $V_{n/n+1}$ <0.15 indicates that n-number of reference genes is sufficient for obtaining reliable results and inclusion of an additional (n+1) control gene is not required.

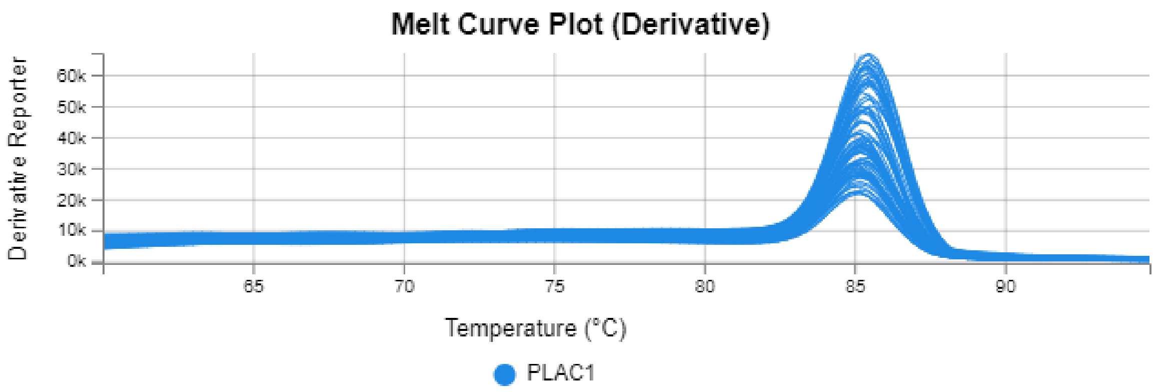
Melt Curve Plot (Derivative)
Derivative Reporter
60k
50k
40k
30k
20k
10k
0k
65
70
75
80
85
90
Temperature (°C)
PLAC1

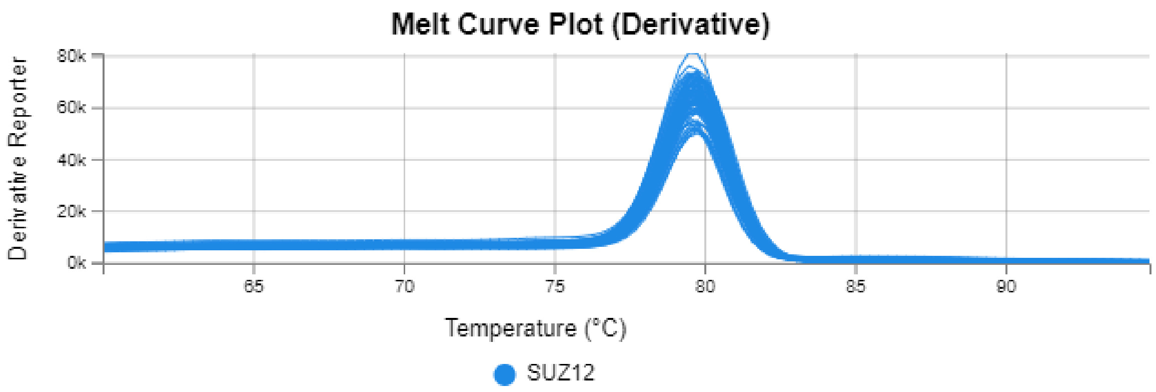
Melt Curve Plot (Derivative)
Derivative Reporter
80k
60k
40k
20k
0k
65
70
75
80
85
90
Temperature (°C)
SUZ12

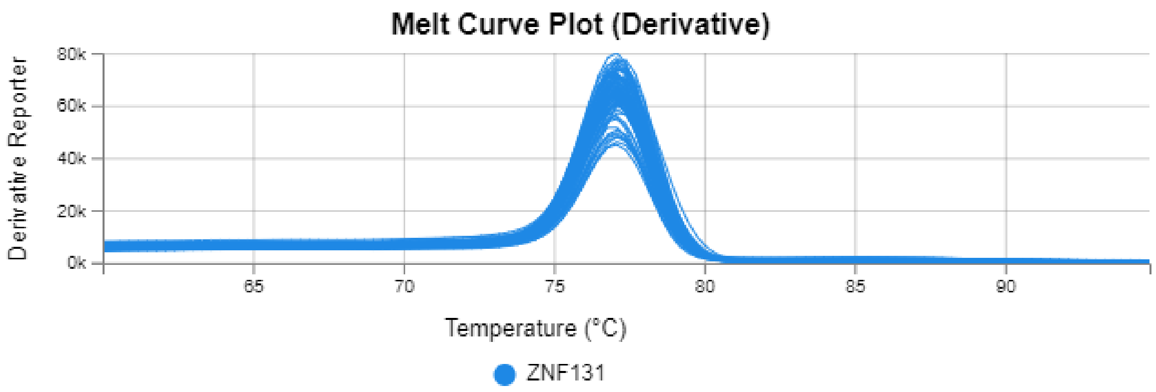
Melt Curve Plot (Derivative)
Derivative Reporter
80k
60k
40k
20k
0k
65
70
75
80
85
90
Temperature (°C)
ZNF131

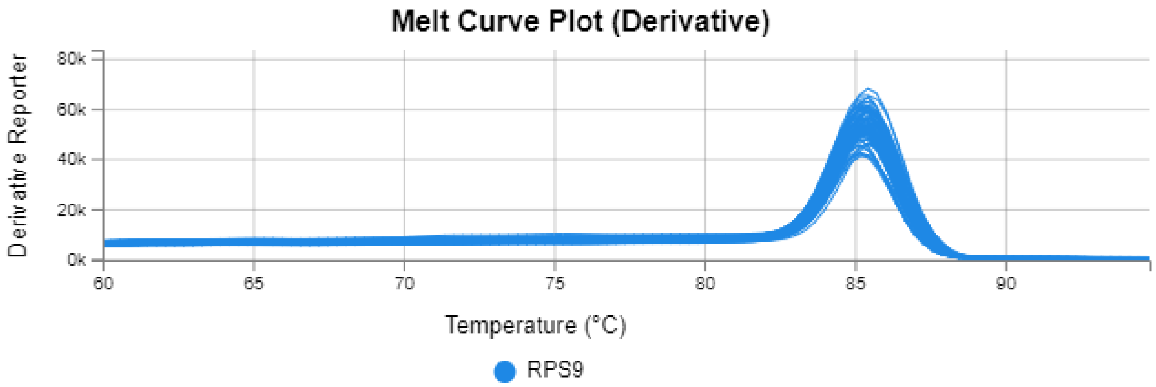
Melt Curve Plot (Derivative)
Derivative Reporter
80k
60k
40k
20k
0k
60
65
70
75
80
85
90
Temperature (°C)
RPS9

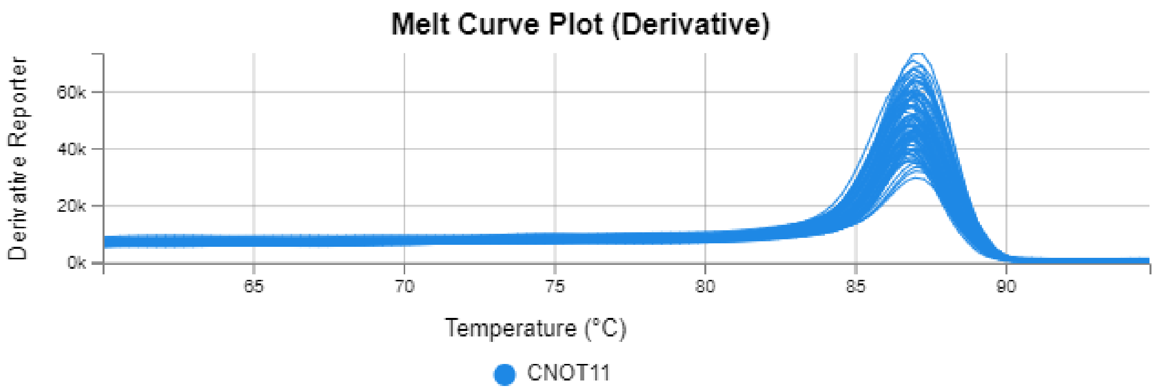

Melt Curve Plot (Derivative)
Derivative Reporter
60k
40k
20k
0k
65
70
75
80
85
90
Temperature (°C)
CNOT11

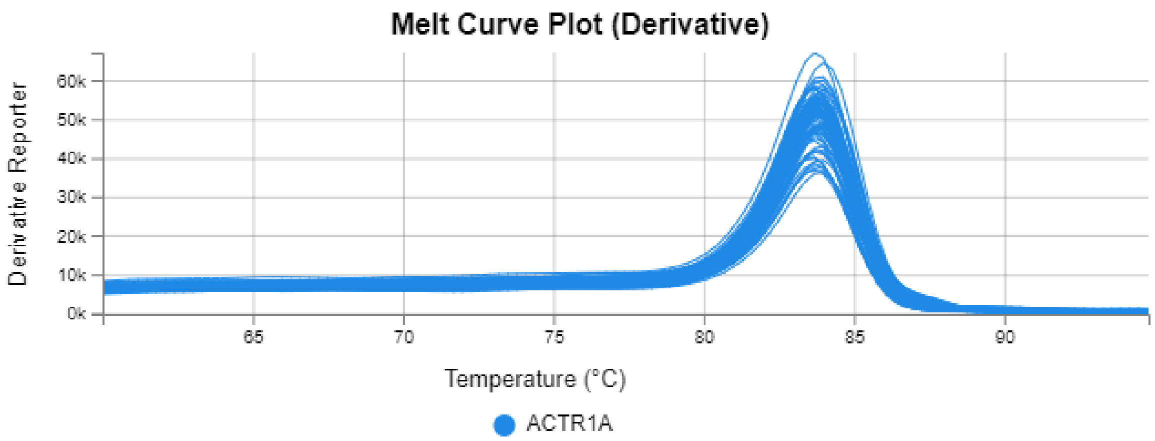

Melt Curve Plot (Derivative)
Derivative Reporter
60k
50k
40k
30k
20k
10k
0k
65
70
75
80
85
90
Temperature (°C)
ACTR1A

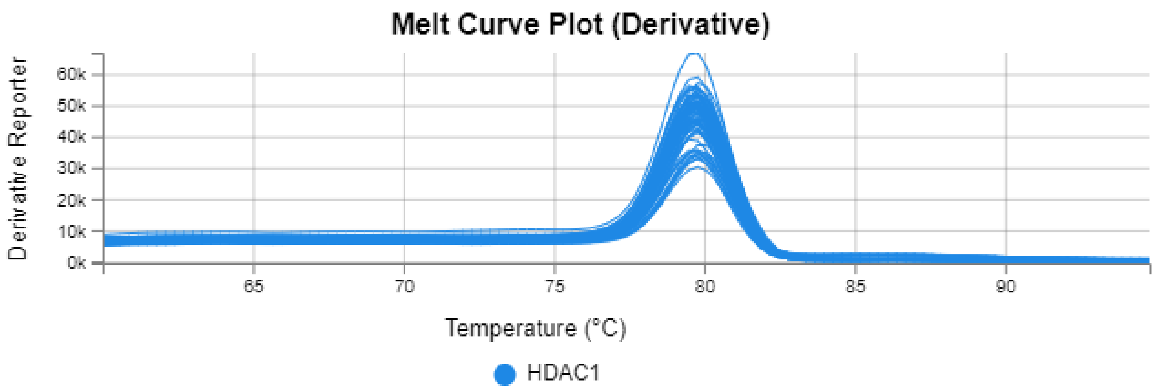

Figure S4. Dissociation curves obtained for gene of interest (*PLAC1*) and candidate reference genes tested in this study.

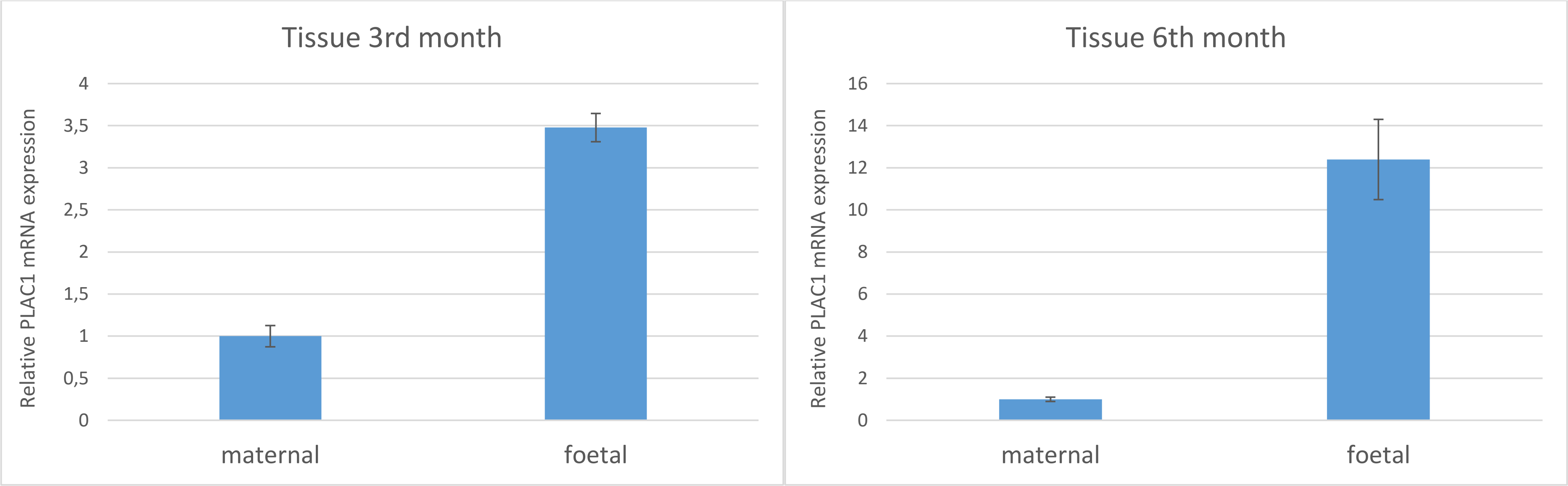

Figure S5. Different expression of PLAC1 mRNA between maternal and foetal part of the placenta in the 3rd and 6th months of pregnancy in cows. Different letters represent statistical significance at $p < 0.01$.
